# Periodontal status among patients with diabetes in Nuuk, Greenland

**DOI:** 10.3402/ijch.v73.26093

**Published:** 2014-12-11

**Authors:** Amanda Lamer Schjetlein, Marit Eika Jørgensen, Torsten Lauritzen, Michael Lynge Pedersen

**Affiliations:** 1 Faculty of Health and Medical Sciences, University of Copenhagen, Copenhagen, Denmark; 2 Steno Diabetes Center, Gentofte, Denmark; 3 Centre for Health Research in Greenland, Southern Denmark University, Odense, Denmark; 4 Section of General Practice, Department of Public Health, Aarhus University, Arhus, Denmark; 5 Queen Ingrid Health Care Centre, Nuuk, Greenland; 6 Greenland Centre for Health Research, Institute of Nursing and Health Science, University of Greenland, Nuuk, Greenland

**Keywords:** diabetes, periodontitis, Greenland, Inuit

## Abstract

**Background:**

Diabetes is becoming more common in the Greenlandic population. Patients with diabetes are more prone to periodontal disease. Periodontal status may have an effect on metabolic control.

**Objective:**

The aim of this study was to estimate the prevalence of periodontitis amongst patients with diabetes in Nuuk, Greenland, and secondly, to observe if dental care was associated with improved periodontal status and metabolic control.

**Study design:**

Observational cross-sectional study and a pilot study of a dental care intervention.

**Methods:**

Sixty-two Greenlandic patients with diabetes were included in the study. Data were collected from the Electronic Medical Records (EMR), in addition to a telephone interview. Patients were offered 3 dental examinations with a 3-month interval. The dental examinations consisted of a full-mouth assessment of number of remaining teeth and assessment of periodontal status. Patients received scaling and root planing, together with information and instructions on oral hygiene. Information on glycated haemoglobin (HbA_1C_) values was collected from the EMR at each dental examination.

**Results:**

In this study, 21.0% (13/62) of patients with diabetes had periodontitis. About 42% had less than 20 teeth. The association between diabetes and periodontitis was known by 20 out of the 62 patients. Over half of the patients had been to a dental examination within the last year. The prevalence of periodontitis decreased significantly from 21.0 to 0% (p<0.001) after 3 dental examinations. No change in HbA_1C_ levels was observed (p=0.440).

**Conclusion:**

Periodontitis was common among patients with diabetes in Nuuk. Dental health status based on Periodontal Screening Index (PSI) and bleeding on probing (BOP) seemed to improve after dental health care, indicating a need for increased awareness among patients and health care professionals. HbA_1C_ levels were not improved among the patients.

While (type 2) diabetes was almost non-existing in Greenland 50 years ago, recent population studies have documented a high prevalence of diabetes affecting around 10% of adults in Greenland, most of them undiagnosed (1). Along with a national diabetes programme aiming to improve diabetes care in Greenland 2008–2011, the prevalence of diagnosed diabetes increased in Greenland (2,3). The diabetes care improved within a few years throughout Greenland. The quality of the diabetes care was described using international accepted diabetes health care indicators focusing on measured HbA_1C_, blood pressure, serum cholesterol, urine, foot and eye examinations (3). Dental care was not included among the selected indicators and the status of dental health and health care among patients with diabetes in Greenland is unknown.

Periodontitis is a common and chronic condition of the tooth-supporting tissue caused by bacterial deposits accumulating on tooth surface and forming dental plaque (4,5). The systemic inflammation associated with periodontitis has been found to aggravate systemic diseases like diabetes (6,7). The prevalence of periodontitis among patients with diabetes in Greenland remains unknown.

Poorly controlled diabetes is in turn associated with bacterial infections, and may influence treatment of periodontitis negatively (8). Success rate with dental implants is thus lower among patients with diabetes. Poorly controlled periodontitis and diabetes may thus mutually reinforce a vicious circle. Both periodontitis and diabetes mellitus are associated with changeable lifestyle factors (4,9,10). Periodontitis is associated with oral hygiene, smoking, malnutrition, obesity, psychological stress and alcohol consumption, and diabetes to hypertension, dyslipidaemia and lack of physical activity, as well as obesity (9–11).

The aim was to estimate the prevalence of periodontitis among Greenlandic patients with diabetes, and secondly, to study if dental care was associated with improved periodontitis status and metabolic control in patients with diabetes.

## Materials and methods

### Study design and period

This study was performed as a cross-sectional study based on dental examinations of patients with diabetes in Nuuk, Greenland. It was designed as a pilot study to examine the possibility of conducting a clinical intervention study in the Greenlandic diabetes population. The study was conducted from June 2013 to February 2014.

### Setting

More than a quarter of the whole population of Greenland lives in the capital Nuuk, a city of 16,818 inhabitants (12). Health care, including dental care, is free to everyone with a permanent address in Greenland. All patients with diabetes and a permanent address in Nuuk are affiliated to Queen Ingrid Health Care Centre. Patients with diabetes are coded with “D” and can be identified using a statistical module within the Electronic Medical Records (EMR).

### Study population

All patients with diabetes aged 20 or above with a permanent address in Nuuk, were identified through the EMR and contacted by telephone. The patients included in the study were Greenlandic patients with diabetes, were permanent residents in Nuuk and were interested in receiving dental examinations. Persons born in Greenland were considered Greenlandic.

### Data collection

Information about gender, age at examination, diabetes duration and glycated haemoglobin (HbA_1C_) was obtained from the EMR at baseline. HbA_1C_ values were thereafter collected from the EMR. Determinations of HbA_1C_ values were accepted if they were measured 6 weeks prior or after the dental examination. A telephone interview was conducted, where questions about the patient's lifestyle was asked. These included questions about weekly sugar intake, alcohol consumption and daily smoking habits. High alcohol intake was defined as 14 units (1 unit equivalent of 12 g of alcohol) or above for women and 21 units or above for men per week. High intake of sugar was defined as sweets or sugary drinks more than 3 times a week. The patients were also asked if they had been to a dental examination within the last year, and if they were aware of the association between diabetic control and periodontal health. In order to pilot a possible future intervention study, the patients were prompted to use the free dental service and offered an appointment for a routine dental examination. In total, the patients were offered 3 appointments for dental examinations, with a 3-month interval.

### Dental examination

The dental examinations were performed by a clinical consultant of oral care educated to examine dental status. The dental examinations consisted of a full-mouth assessment of number of remaining teeth and assessment of periodontal status. The periodontal status was defined by Periodontal Screening Index (PSI) using a WHO probe (13–16). PSI was defined as follows: Score 0=healthy, score 1=bleeding, score 2=supra-/subgingival dental calculus, score 3=probing depths from 3.5 mm to max. 5.5 mm, score 4=probing depths greater than 5.5 mm (15). “No periodontitis” was thus defined as a score below 3, and “periodontitis” as a score of 3 or above (14). In addition to PSI score, bleeding on probing (BOP) was described as a solitary variable and presence of plaque was recorded whenever soft or hard plaque was observed in the dental examination. Furthermore, all patients received scaling and root planing and information and instructions on oral hygiene. If caries or other dental conditions in need of dentist intervention were found, the patient was referred to a dentist.

### Analysis and statistics

The measurement of HbA_1C_ was conducted at The Central Laboratory at Queen Ingrid Hospital in Nuuk and analysed by the principles of an ion-exchange high-performance liquid chromatography. The apparatus used was G8 HPLC Analyzer^®^ (Tosoh Bioscience, Stuttgart, Germany). The Central Laboratory at Queen Ingrid Hospital is a member of the Danish Quality Control System. A confirmed value of HbA_1C_ at or above 6.5% (48 mmol/mol) is used as a diagnostic for diabetes mellitus in Greenland, as recommended by the World Health Organization (17).

Statistical analysis was performed using SSPS 21.0 software. Variables were described using medians and quartiles (Q1–Q3). Proportions were calculated with 95% confidence intervals (95% CI). Medians were compared using Mann-Whitney U-test. Proportions were compared using Chi-square test. A P-value below 0.05 was used as a significance level.

### Ethics

All participants were informed about the study by telephone and again on arrival to the dental clinic. This study was conducted using data stored in the EMR and by prompting patients to use the free dental care already available to all Greenlanders. Information was given and informed consent was requested. The Ethics Review Committee of Greenland approved the project.

## Results

### Study population

**Fig. 1.**
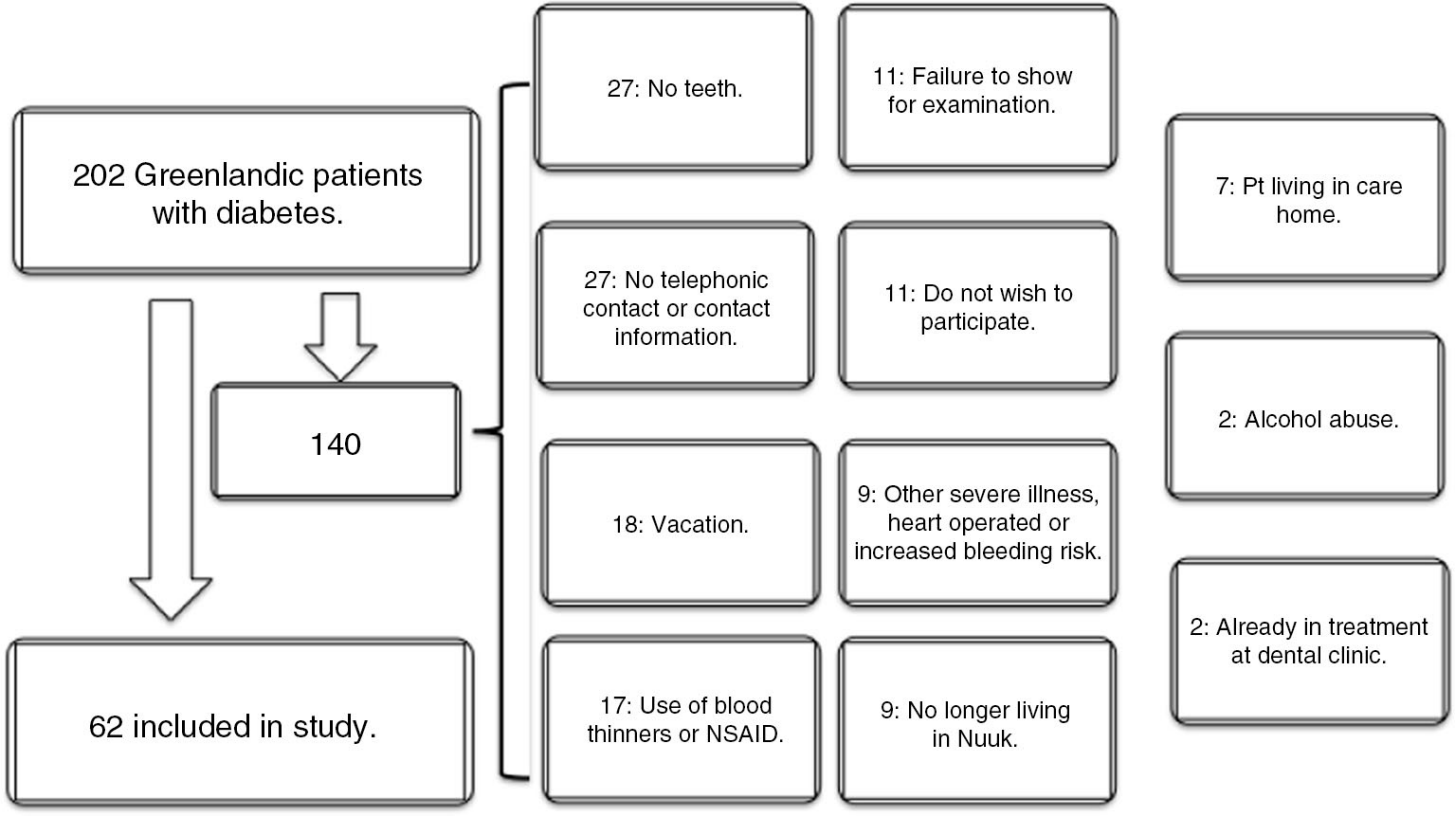
Flowchart showing reasons for exclusion of the study.

A total of 202 Greenlandic patients with diabetes in Nuuk constituted the study population. Of those, 140 were excluded from the pilot study. Reasons for exclusion are given in Figure 1. The major causes of exclusion were treatment with blood thinning drugs, 12.1% (n=17), no teeth, 19.3% (n=27), and difficulty in gaining telephone contact, 19.3%. The remaining 62 patients made up our study group. All 62 patients attended the first dental examination, 53 attended the second, and the final examination was attended by 49 of the original 62 patients. Results were available for 58 of the 62 patients in connection with the first dental examination, 48 in connection with the second and finally 45 patients in connection with the last dental examination. Of the 62 patients, 13 had their medication adjusted during the observation period. Thus, 11 (18%) patients had their glucose-lowering drugs therapy intensified. TD2 was the most common type of diabetes, with 57 patients. The remaining 5 had TD1.

At the first dental examination 1 patient had a score of 0; 11 patients had a score of 1; 37 patients had a score of 2; 13 patients had a score of 3 and no patients had a score of 4. There was no significant difference between men and women (p=0.182). Thus, at baseline 21.0% (95% CI: 11–31) of patients in our study population had periodontitis. Baseline characteristics of the study population and periodontitis status are shown in Table I. Twenty-six (41.9%) of the patients had less than 20 teeth. The association between diabetes and dental health was known by 20 patients. More than half of the patients (56.5%) had a dental examination within the last year. A total of 36 patients were referred to a dentist at the first dental examination, due to caries or problems with tooth fillings. Smoking, high sugar intake and no alcohol consumption were not associated with periodontitis status. No difference in metabolic control was observed among patients with and without periodontitis. Periodontal health and metabolic control for each dental examination are listed in Table II. After the dental examinations the overall prevalence of periodontitis was reduced significantly from 21.0 to 0% (p<0.001). BOP was also reduced significantly from 92 to 33% (p<0.001). The presence of plaque was observed in 54.8% (34/62) of the patients at first examination and 36.7% (18/49) at the third (p=0.058). However, no difference in median HbA_1C_ level was observed between first and last examination (p=0.440).

**Table I:** Baseline characteristics of the study population, median, (Q1–Q3) or percentage

	Total	No periodontitis (PSI<3)	Periodontitis (PSI≥3)	
	(n=62)	(n=49)	(n=13)	p
Age, years	57 (51–60)	57 (51–61)	56 (53–59)	1.000
Men, n (%)	28 (45.2)	25 (51.0)	3 (23.1)	0.182
No. of teeth, median	21 (15–26)	22 (15–26)	20 (16–21)	0.082
Fewer than 20 teeth, n (%)	26 (41.9)	20 (40.8)	6 (46.2)	0.729
No. years with diabetes	3.5 (1–8)	4 (1–10)	3 (1–5)	1.000
HbA1c,%^a^	7.1 (6.4–8.2)	7.0 (6.4–8.3)	7.3 (6.8–7.5)	1.000
HbA1c, mmol/mol^a^	54 (46–66)	53 (46–67)	56 (51–58)	1.000
Daily smoking, n (%)	33 (53.2)	24 (49.0)	9 (69.2)	0.193
No use of alcohol, n (%)	17 (27.4)	16 (33.7)	1 (7.7)	0.073
High intake of sugar, n (%)	21 (33.9)	17 (34.7)	4 (30.7)	0.790
Awareness dental health and diabetes, n (%)	20 (32.3)	15 (30.6)	5 (38.5)	0.590
Dental examination within 1 year, n (%)	35 (56.5)	26 (53.1)	9 (69.2)	0.296

a n=58.

**Table II:** Periodontal health, bleeding on probing and metabolic control at the 3 dental examinations

	First examination			Second examination			Third examination		
	
	Total n=62	Men n=28	Women n=34	Total n=53	Men n=23	Women n=30	Total n=49	Men n=22	Women n=27
No periodontitis PSI<3,%	79 (49/62)	71.4 (20/28)	85.3 (29/34)	96.2 (51/53)	100 (23/23)	93.3 (28/30)	100 (49/49)	100 (22/22)	100 (27/27)
Bleeding on probing,%	92 (57/62)	96 (27/28)	88 (30/34)	55 (29/53)	74 (17/23)	40 (12/30)	33 (16/49)	45 (10/22)	22 (6/27)
	n=58	n=25	n=35	n=48	n=20	n=28	n=45	n=18	n=27
HbA1c (%) Median (Q1–Q3)	7.1 (6.2–8.1)	7.4 (6.4–9.2)	6.8 (6.5–7.8)	6.9 (6.5–7.9)	7.4 (6.4–8.5)	6.7 (6.5–7.5)	7.0 (6.6–8.3)	7.6 (6.5–8.6)	6.7 (6.6–7.8)
HbA1c mmol/mol – Median (Q1–Q3)	54 (44–65)	57 (46–77)	51 (48–62)	52 (48–63)	57 (46–69)	50 (48–58)	53 (49–67)	60 (48–70)	50 (49–62)

## Discussion

The prevalence of periodontitis among patients with diabetes was 21.0% at inclusion in the study. This was markedly reduced to 0% at the end of the study, suggesting a positive effect of dental examinations by a clinical consultant. Also, the significant reduction in BOP after the dental examinations further indicates a reduction in periodontal inflammation. However, no significant improvement in metabolic control was observed within 6 months.

A notable high number of patients (27/140) were excluded due to lack of teeth. Furthermore, several (26/62) patients had less than 20 teeth indicating poor dental health. This is in accordance with general poor dental health status among children and adolescents in Greenland, reported 10 years ago (18). At all ages, high proportions of children had severe patterns of dental caries. The dental status was significantly worse than in Denmark (18). Thirty-two per cent of the included patients were aware of an association between dental health and diabetes and 56% had actually visited a dental clinical consultant of oral health or a dentist within the last year. However, increased awareness of dental health may be possible. Awareness of the association has thus been reported higher. Weinspach et al. reported that 44% of patients were aware of the association in the German population (6). Weinspach et al. also reported that diabetes patients were 3 times more likely to develop periodontitis, especially T2D patients who are more susceptible to poor periodontal health (6).

### Other studies

At inclusion, 21.0% of all patients had periodontitis in this study. American Indians and Alaska Natives with diabetes were found to have a prevalence of periodontitis of 34% (19). This prevalence was higher than for non-diabetics. A similar finding was reported in Roberts-Thomson et al. study in an aboriginal population in Australia, where diabetes patients had a higher severity score of periodontitis than non-diabetics. They found a prevalence score of periodontitis amongst all subjects at 11.9% (20). A large population study in the United States found that 64% of patients over 65 years of age had moderate or severe periodontitis (21), probably due to higher age in this population. The prevalence of periodontitis in the present study is quite similar to the prevalence reported for American Indians and Alaska Natives and aboriginals (19,20).

In our study, we observed a marked reduction in periodontitis following regular dental visits, indicating a possible approach to improve periodontal health in patients with diabetes. This is in agreement with the findings in other studies (22,23), where a positive effect on periodontal health after full-mouth scaling and root planing has been reported. In light of this, the joint EFP/AAP workshop on periodontitis and systemic diseases, offer guidelines to physicians and dentists on how to treat patients with diabetes and periodontitis (24).

However, we failed to document a decrease in HbA_1C_ levels after 3 dental examinations, even though diabetic medication was adjusted for 18% of patients during the study period.

Other studies have reported a lowering of HbA_1C_ when periodontal health was improved (22–25). Thus, Sgolastra et al. found that scaling and root planing in T2D patients with chronic periodontitis significantly lowered HbA_1C_ levels (25). In addition, Kiran et al. found that HbA_1C_ levels improved significantly with improvement of periodontal health (23). Moeintaghavi et al. also found a decrease of HbA_1C_ with non-surgical treatment of periodontitis, although this was not significant (22). A recent Cochrane review on the correlation between diabetes and periodontitis was conducted in 2010, concluding that treatment of periodontal disease had a beneficial, albeit limited, effect on glycaemic control (26). The failure of this study to document an improvement in HbA_1C_ levels, may be due to the limitations listed below in Strengths and limitations.

However, in a multi-centred randomized controlled trial with 514 participants, non-surgical periodontal treatment showed no improvement on glycaemic control (27). Similarly, 154 T2D patients in a randomized controlled trial from 2014 in a Hispanic population in the United States found no significant reduction in HbA_1C_ levels when patients with T2D were given non-surgical periodontal therapy (28). Both studies did, in turn, find a significant improvement in periodontal status. This is in accordance with the results of this study.

### Strengths and limitations

The main strength of this study was that all patients with diagnosed diabetes in Nuuk were included. Furthermore, diabetes and the correlation with periodontitis have never been examined in Greenland before. The main limitation was the low number of patients included in the study and the lack of a control group. A possible positive effect on HbA_1c_ may have been found if compared to a control group. In addition, around 20% (13/62) of the patients did not participate in the final examination, thus further limiting the power of the study. The observation period was quite short. This limits the possibility to conclude on long-term effects. Also, HbA_1C_ values at inclusion were performed in summertime in contrast to final HbA_1C_ values collected in the wintertime. This seasonal variation may have masked an effect on metabolic control since summer values are reported to be lower than winter values (29). Tseng et al. found that HbA_1C_ had significant seasonal fluctuations, especially in regions with large differences between summer and winter temperature, as in Nuuk (29).

Furthermore, classification of periodontitis based on PSI alone was not optimal. Although the method is widely used, both in research settings and as an element in routine initial dental examinations, limitations exist. To visualize proximal bone loss, radiographic methods were required, and it cannot be excluded that some of the patients classified with periodontitis had only pseudopockets. Also, the lack of a real plaque score was suboptimal. However, the reduction in PSI was accompanied by a significant reduction in BOP, indicating reduced inflammation.

## Conclusion

This pilot study was conducted to evaluate the feasibility of a study evaluating whether improved periodontitis care could improve HbA_1c_ levels. Our pilot study indicates that the number of patients needed for such a study would be all too big and the improvement in HbA_1c_ would most likely be clinically irrelevant.

However, some lessons can be drawn from this study: Periodontitis was common among patients with diabetes in Nuuk. Dental health status based on PSI and BOP seemed to be improved after dental health care among most of the patients with diabetes included in this study, indicating a need for increased awareness of dental health among patients and health care professionals.
